# Involvement of dopamine loss in extrastriatal basal ganglia nuclei in the pathophysiology of Parkinson’s disease

**DOI:** 10.3389/fnagi.2014.00087

**Published:** 2014-05-13

**Authors:** Abdelhamid Benazzouz, Omar Mamad, Pamphyle Abedi, Rabia Bouali-Benazzouz, Jonathan Chetrit

**Affiliations:** ^1^Institut des Maladies Neurodégénératives, Université Bordeaux Segalen, UMR 5293Bordeaux, France; ^2^CNRS, Institut des Maladies Neurodégénératives, Université Bordeaux Segalen, UMR 5293Bordeaux, France; ^3^Faculté des Sciences, Equipe Rythmes Biologiques, Neurosciences et Environnement, Université Mohamed V-AgdalRabat, Morocco; ^4^Institut Interdisciplinaire des Neurosciences, Université Bordeaux Segalen, UMR 5297Bordeaux, France

**Keywords:** dopamine, extrastriatal dopamine, basal ganglia, globus pallidus, subthalamic nucleus, Parkinson’s disease

## Abstract

Parkinson’s disease (PD) is a neurological disorder characterized by the manifestation of motor symptoms, such as akinesia, muscle rigidity and tremor at rest. These symptoms are classically attributed to the degeneration of dopamine neurons in the *pars compacta* of substantia nigra (SNc), which results in a marked dopamine depletion in the striatum. It is well established that dopamine neurons in the SNc innervate not only the striatum, which is the main target, but also other basal ganglia nuclei including the two segments of globus pallidus and the subthalamic nucleus (STN). The role of dopamine and its depletion in the striatum is well known, however, the role of dopamine depletion in the pallidal complex and the STN in the genesis of their abnormal neuronal activity and in parkinsonian motor deficits is still not clearly determined. Based on recent experimental data from animal models of Parkinson’s disease in rodents and non-human primates and also from parkinsonian patients, this review summarizes current knowledge on the role of dopamine in the modulation of basal ganglia neuronal activity and also the role of dopamine depletion in these nuclei in the pathophysiology of Parkinson’s disease.

## Introduction

Parkinson’s disease (PD) is a neurological disorder characterized by the manifestation of motor symptoms such as akinesia, muscle rigidity and tremor at rest. These motor deficits are classically attributed to the degeneration of dopamine neurons in the *pars compacta* of substantia nigra (SNc), which result in a marked dopamine depletion in the striatum, the primary projection region of the SNc. Furthermore, it is well established now that dopamine neurons in the SNc innervate not only the striatum but also other basal ganglia nuclei including the two segments of globus pallidus, the external part (GPe in primate, the equivalent of GP in rodents) and the internal part (GPi in primate, the equivalent of entopeduncular nucleus in rodents), as well as the subthalamic nucleus (STN; Smith and Villalba, 2008). Dopamine has been shown to modulate the neuronal electrical activity of all these basal ganglia nuclei (Rommelfanger and Wichmann, 2010).

Dopamine cell degeneration in the pathophysiology of PD is considered as the main hallmark of the disease (Agid and Blin, 1987; Hornykiewicz, 1998). Indeed, dopamine depletion by stereotaxic injection of 6-hydroxydopamine (6-OHDA) in the rat or by systemic injections of 1-methyl-4-phenyl-1,2,3,6-tetrahydropyridine (MPTP) in the non-human primate resulted in alterations of the firing rate and/or patterns of GPe, GPi and STN neurons. The tonic regular pattern in the normal condition changed toward a pathological exaggerated burst firing with oscillations after dopamine cell lesions in the substantia nigra (SNc; Albin et al., 1989; DeLong, 1990; Bergman et al., 1994; Wichmann et al., 1994; Boraud et al., 1998; Ni et al., 2000, 2001b; Magill et al., 2001; Breit et al., 2007; Rivlin-Etzion et al., 2010). Similar bursty pattern has been reported in PD patients when microrecordings have been done during surgery for the implantation of deep brain stimulation electrodes (Hutchison et al., 1998; Benazzouz et al., 2002). According to the classical model of the anatomo-functional organization of the basal ganglia, the pathological activity recorded in basal ganglia nuclei has been identified as a consequence of dopamine depletion in the striatum (Albin et al., 1989).

In normal physiological conditions, dopamine has long been known to be a crucial neuromodulator of striatal processing of cortical informations carried by glutamatergic synapses on medium spiny neurons, which represents the principal projection neurons of the striatum. Dopamine excites medium spiny neurons of the “direct” pathway through dopamine D1 receptors, while it inhibits striatal neurons of the “indirect” pathway through dopamine D2 receptors (Alexander and Crutcher, 1990; Surmeier et al., 2007). In the context of PD, studies of the neuronal activity in the basal ganglia of MPTP monkeys and 6-OHDA rat models of the disease suggested that the direct and the indirect pathways are differentially affected by the loss of dopamine in the striatum. The GABAergic inhibitory direct striato-GPi pathway becomes underactive, whereas the GABAergic projection from the striatum to the GPe of the indirect pathway becomes overactive, leading to the reduced activity along the inhibitory GPe-GPi and GPe-STN pathways. Thus, it is suggested that exaggerated oscillatory bursts in STN and in GPi may have been secondary to tonic disinhibition of both structures from GPe after loss of dopamine in the striatum. However, the role of dopamine depletion in these extrastraiatal basal ganglia nuclei in the pathophysiology of PD is still not clearly defined. Nevertheless, in view of the demonstrated physiologic actions of dopamine on pallidal and STN neuronal activity as well as the effects on motor behavior of local injection of dopamine drugs, it is assumed that the loss of pallidal and subthalamic dopaminergic control would contribute to the motor symptoms in PD (Rommelfanger and Wichmann, 2010; Wilson and Bevan, 2011).

The pallidal complex and the STN are innervated by nigral dopamine fibers, by separate fiber system and also by collaterals of nigrostriatal fibers. This has been shown in rodents (Lindvall and Bjorklund, 1979; Debeir et al., 2005; Anaya-Martinez et al., 2006), in non-human primate (Nobin and Bjorklund, 1973; Parent and Smith, 1987; Lavoie et al., 1989; Parent et al., 1989; François et al., 1999; Hedreen, 1999; Jan et al., 2000) and in human brains (Nobin and Bjorklund, 1973; Cossette et al., 1999; François et al., 1999; Jan et al., 2000). Dopamine acts via five receptor subtypes subdivided into two receptor families: D1 (D1 and D5 subtypes) and D2 (D2, D3 and D4 subtypes). All are prototypic of G-protein-coupled receptors with dopamine D1 receptors being positively linked to adenylate cyclase and D2 receptors had negative coupling to the enzyme (Kebabian and Calne, 1979). A large number of experimental studies reported that functional dopamine receptors are expressed in the striatum and also in the GPe, GPi and STN and that dopamine modulates their neuronal activity through a variety of mechanisms via pre- and post-synaptic sites (Smith and Villalba, 2008; Rommelfanger and Wichmann, 2010).

Dopamine, through D1 and D2 family receptors, in the pallidal complex and the STN may modulate the motor circuit and consequently dopamine depletion in these structures may play a role in the pathophysiology of PD. Lesions of dopamine neurons in the SNc in rodents and monkeys have been shown to reduce dopamine levels in GPe, GPi and STN in addition to the striatum (Parent et al., 1990; François et al., 1999; Jan et al., 2000; Fuchs and Hauber, 2004).

## Dopamine depletion in the globus pallidus

Rajput et al. (2008) have recently reported a marked loss of dopamine in the GPe (−82%) of PD patients with a severe loss of dopamine in the caudate (−89%) and the putamen (−98.4%). Based on the conclusions of a previous experimental study (Pifl et al., 1991), the authors suggested that pallidal dopamine participates in the functional compensation against the severe loss of dopamine in the striatum at the early stage of the disease. It has been shown that in the MPTP-treated primate model of parkinsonism in which the animals with stable parkinsonian symptoms showed a marked pallidal dopamine depletion, asymptomatic animals showed normal pallidal dopamine levels but had very marked striatal dopamine deficit (Pifl et al., 1991). Similarly, imaging studies using positron emission tomography in PD patients reported that while patients with severe advanced stage of the disease had significantly reduced 18F-dopa uptake in the striatum, GPe and GPi, patients at mild stage of the disease demonstrated a severely reduced 18F-dopa uptake in the striatum but normal uptake in GPe and GPi (Whone et al., 2003; Pavese et al., 2011). Furthermore, an increase in 18F-dopa uptake in GPi has been reported in early stage PD (Rakshi et al., 1999; Whone et al., 2003; Moore et al., 2008; Pavese et al., 2011). Together, these studies postulate that dopamine plays a key role in the compensatory up-regulation of the nigro-pallidal dopamine projection in the early stages of PD representing a compensatory adaptive mechanism to preserve functionality. In contrary, dopamine depletion in the two pallidal segments (GPe and GPi) may participate in the aggravation of motor symptoms in the late stages of PD.

Studies on the expression of dopamine receptors in the pallidal complex of parkinsonian brains reported conflicting data. While some studies found no difference in dopamine D1R expression in the GPe and GPi (Rinne et al., 1985; Cortés et al., 1989), others found dopamine D1R expression unchanged in the GPi and decreased in GPe (Hurley et al., 2001). Dopamine D2 receptors, including D3 receptors, were unchanged in both GPe and GPi (Bokobza et al., 1984; Cortés et al., 1989; Ryoo et al., 1998). The absence of changes in the expression of D1 and D2 receptors can be explained by the fact that patients were under dopaminergic medication before death and that the treatment is likely to normalize the expression of these receptors. This may be true for the striatum but not for the pallidal complex as in MPTP monkeys, the expression of dopamine D3 receptors was reduced in the caudate nucleus but not in the GPe and GPi and that L-Dopa treatment normalized the hypoexpression of dopamine D3 receptors in the caudate nucleus and increased to a level higher than normal in GPi without any change in the GPe (Bézard et al., 2003).

The contribution of pallidal dopamine in the pathophysiology of PD has also been demonstrated in rodents. Activation of D1 or D2 dopamine receptors in the GP induced movement facilitation (Sañudo-Peña and Walker, 1998). In contrast, local blockade of D1 and/or D2 receptors by intra-pallidal infusions of specific antagonists induced akinesia in rats (Hauber et al., 1998; Hauber and Lutz, 1999). Similarly, in rats bearing a unilateral 6-OHDA lesion, it has been shown that blockade of either dopamine D1 or D2 receptors reduced apormorphine-induced turnings and that dopamine infusion into the GP improved motor deficits in the same animal model (Galvan et al., 2001). Furthermore, we have recently shown that intra-pallidal injection of 6-OHDA produced deficits of dopaminergic transmission that caused asymmetrical motor impairment and reduction of locomotor activity in the rat (Bouali-Benazzouz et al., 2009; Abedi et al., 2013). Together, these studies provide arguments that dopamine transmission within the globus pallidus is necessary to achieve motor control and that its lack plays a role in the pathophysiology of parkinsonian motor symptoms, in addition to dopamine depletion in the striatum.

## Dopamine depletion in the subthalamic nucleus

STN holds a pivotal position in basal ganglia circuitry exerting an excitatory drive on the output structures of the system (Albin et al., 1989; Alexander and Crutcher, 1990). The STN has been shown to play a key role in motor control, as its hyperactivity with oscillatory bursts has been associated to parkinsonian motor deficits. The motor symptoms can be reversed by selective STN lesion or high frequency stimulation (Bergman et al., 1990; Benazzouz et al., 1993; Limousin et al., 1995; Krack et al., 2003). Several studies have suggested the implication of SNc-STN dopaminergic projection in the pathophysiology of PD. Thus, bilateral infusions of D1 but not D2 receptor antagonists into the STN induced catalepsy in normal rats (Hauber, 1998) and that activation of D1 receptors resulted in orofacial dyskinesias in normal and dopamine-depleted rats (Parry et al., 1994; Mehta et al., 2000). From these studies it is suggested that dopaminergic agents acting at D1 receptors have stronger functional and behavioral effects than agents acting at D2 receptors. Several studies have shown that dopamine is reduced in the STN in experimental Parkinsonism and also in patients with PD (Pifl et al., 1990; Hornykiewicz, 1998; François et al., 2000) and such dopamine loss in the STN may contribute to increase abnormal neuronal activity. Accordingly, selective lesions of the dopaminergic fibers located in the STN, by intra-subthalamic infusion of 6-OHDA, resulted in contralateral muscle rigidity and ipsilateral turning in response to systemic administration of DL-methamphetamine (Flores et al., 1993). These motor deficits could be explained by the fact that 6-OHDA injection into the STN resulted in retrograde degeneration of dopamine cell bodies in the lateral part of the SNc (Ni et al., 2001a) and was at the origin of the significant increase in the percentage of STN neurons exhibiting burst pattern (Ni et al., 2001b). These results provide evidence that the degeneration of SNc-STN dopaminergic projections plays, at least in part, a role in the development of the pathological burst pattern of STN neurons and therefore to the manifestation of PD-like motor deficits. In the same 6-OHDA rat model, it has been shown that lesions of the SNc dopaminergic cells increased the level of dopamine D2 receptor mRNA, decreased D3 receptor mRNA levels, and did not induce significant changes in Dl receptor mRNA in the STN (Flores et al., 1999). Furthermore, several studies have shown that dopamine D5 receptors, which display a high agonist-independent constitutive activity *in vitro* (Tiberi and Caron, 1994; Demchyshyn et al., 2000), are located in the STN and are able to potentiate burst firing in STN neurons in *in vitro* rat brain slices (Baufreton et al., 2003, 2005). These authors suggested that in the parkinsonian state, the reduction of dopaminergic transmission in the STN results in a lack of activation of dopamine D2 receptors, and as D5 receptors are constitutively active even in the absenc of dopamine, they contribute to the development of burst discharges of STN neurons (Baufreton et al., 2005). This assumption has been demontsrated by our recent *in vivo* study, in which we have shown that local microinjection of an inverse agonist of D5 receptors, flupenthixol, reduced burst activity of STN neurons and therefore improved the motor deficits in the 6-OHDA rat model of PD (Chetrit et al., 2013). Moreover, STN dopaminergic afferents have also been suggested to play a relevant role in the expression of dyskinesias. Indeed, dopamine depletion in the STN attenuated levodopa-induced dyskinesia in rats bearing a concomitant lesion of the nigrostriatal pathway (Marin et al., 2013).

## Conclusion

While the degeneration of the dopaminergic nigrostriatal pathway is the hallmark of PD, there is strong evidence about the key role played by dopamine loss at extrastriatal sites, especially in the pallidal complex and the STN, in the pathophysiology of the disease. Activation of dopamine receptors in these important basal ganglia nuclei modulates their neuronal activity, and consequently participates in the beneficial effects, and may be in the adverse effects, of the classical dopaminomimetic antiparkinsonian drugs. Together, we can assume that dopamine transmission within the globus pallidus and the STN is necessary to achieve normal motor control.

## Conflict of interest statement

The authors declare that the research was conducted in the absence of any commercial or financial relationships that could be construed as a potential conflict of interest.
